# Multi-Centre Study of Progression Factors and Intravesical Recurrence in Patients with Urothelial Carcinoma of the Upper Urinary Tract

**DOI:** 10.3390/diagnostics14222491

**Published:** 2024-11-07

**Authors:** Lucía García-Morales, Francisco Javier Contreras-Matos, Ana Blanca-Pedregosa, Alejandro Mellado-Castillero, Juan Pablo Campos-Hernández, María Fernanda Lara, Ignacio Puche-Sanz, Enrique Gómez-Gómez

**Affiliations:** 1 Urology Department, Reina Sofía University Hospital, Maimonides Institute of Biomedical Research of Cordoba (IMIBIC/UCO), Avda. Menéndez Pidal s/n, 14004 Córdoba, Spain; lugarmo18@gmail.com (L.G.-M.);; 2 Department of Urology, Virgen de las Nieves University Hospital, Biosanitary Research Institute of Granada (IBS), 18071 Granada, Spain; 3 Institute of Urological Surgery, 29007 Málaga, Spain; 4 Faculty of Medicine, University of Málaga (UMA), IBIMA, BIONAND, 29010 Málaga, Spain; fer76lc@hotmail.com

**Keywords:** urothelial carcinoma, disease progression, intravesical recurrence, risk factors

## Abstract

Background/Objectives: A retrospective analysis was conducted to identify factors associated with disease progression and intravesical recurrence (IVR) in a multi-centre cohort of patients with upper urinary tract urothelial carcinoma (UTUC) treated surgically between 2015 and 2021. Methods: Progression-free survival (PFS) and IVR-free survival were evaluated using a Kaplan–Meier survival curve and a Log-Rank test. Prognostic factors for progression and IVR were analysed using Cox logistic regression analysis. Results: A total of 170 patients were analysed. Up to 32.9% developed progression within 65.64 ± 3.44 months. Multivariate analysis showed that pT (HR 2.9, 95%CI 1.54–5.48, *p* = 0.01), margin status (HR 2.89, 95%CI 2.88–57.68, *p* = 0.01), and lymphovascular involvement (HR 7.97, 95% CI 1.43–44.42, *p* = 0.02) were independent risk factors for PFS. Up to 25.9% of patients presented with IVR at a mean time of 68.33 ± 3.59 months. A previous diagnosis of bladder cancer (BC) (HR 3.73, 95% CI 1.24–11.22, *p* = 0.02) and the non-invasive appearance of the tumour on computed tomography were significant risk factors for IVR (HR 0.23, 95% CI 0.05–0.95, *p* = 0.03). Conclusions: pT stage, margin involvement, and lymphovascular involvement were independent risk factors for UTUC progression. The main risk factor for presenting with IVR after UTUC was a previous diagnosis of BC.

## 1. Introduction

Urothelial carcinoma of the upper urinary tract (UTUC), which includes lesions from the renal calyces to the distal ureter, comprises 5–10% of urothelium tumours [1]. UTUC presents an annual incidence of approximately 2 cases per 100,000 people. This rate has increased in recent decades because of better detection techniques [2,3].

Some predictors of survival after radical nephroureterectomy (RNU) have been studied, including histological grade, lymphovascular invasion, pathological TNM stage, the presence of concomitant carcinoma in situ (CIS), and multifocality [4]. The survival of patients with UTUC has not improved significantly over time, and up to 30% of patients experience disease recurrence and cancer-specific death [5].

An important cause of concern for patients with UTUC is intravesical recurrence (IVR) after RNU, which occurs in 15–50% of cases [6,7]. IVR has a significant psychological effect on the patient, affecting their quality of life, and also has a considerable economic impact [4].

Our objective was to study different predictors of risk of progression and IVR in a multi-centre cohort to facilitate the optimisation of the diagnosis, management and treatment of these patients.

## 2. Materials and Methods

### 2.1. Patients

This study involved a retrospective analysis of a multi-centre cohort of 170 patients with UTUC confirmed by biopsy and surgically treated at two major hospitals between 2015 and 2021 (ethic approval number [6/21]). Patients treated by RNU were included in most cases, but so were those who had undergone simple nephrectomies, ureterectomies, and endoscopic treatment. The choice of surgical approach depended on the characteristics of the patient and the preferences of the surgeon, as well as the approach to the distal ureter, which was also at the discretion of the main surgeon. Intravesical postsurgical chemotherapy was only administered to a minor group of patients as the technique was not a local common practice in clinical use at the time period of patient recruitment. The patients’ follow-up was carried out according to routine clinical practice, mainly based on endoscopy and CT scans.

### 2.2. Variables Under Study

The clinicopathological variables, variables associated with diagnosis and treatment, and anatomopathological variables were described. A previous diagnosis of BC and previous UTUC were defined as no/yes, and a synchronous diagnosis was defined as bladder and upper urothelial tract cancer diagnosed at the same time. The occurrence of progression was defined as local (defined as recurrence in the ureteral remnant or in the ipsilateral renal lodge, excluding IVR), nodal, visceral (excluding urothelial tissue), or bone progression on follow-up CT scan, and IVR was evaluated by cystoscopy and biopsy.

### 2.3. Statistical Analysis

A descriptive analysis was carried out, evaluating the quantitative variables through the mean and SD, and the qualitative variables with the absolute and relative values.

Prognostic factors for progression and IVR were evaluated in these patients using multivariate Cox regression analysis. The PFS and IVR analyses were performed using a Kaplan–Meier survival curve. All calculations were performed with the statistical package IBM^®^ SPSS^®^ statistics v-21. Those with a *p* value of < 0.05 were considered statistically significant.

This study was reviewed and approved by “CEIM/CEI Provincial de Granada, Ethics Committee”, approval number [6/21].

## 3. Results

### 3.1. Descriptive Study

A total of 170 patients were analysed. The main descriptive variables are shown in Table 1. The mean age was 69 ± 11 years, the majority being men (74.1%) and active or former smokers (66.4%), with a mean BMI of 28.92 ± 4.5 kg/ m^2^. A total of 24.1% patients had experienced previous BC, and 11.8% of them a synchronous diagnosis.

Most patients were diagnosed by CT or CT urogram and presented with a mean tumour size of 3.5 cm ± 2.0. The most frequent location was pyelocalyceal (41.2%), and 31.8% were multifocal. Diagnostic ureteroscopy was performed in 27.6% of the patients, associated with a biopsy in 12.3%.

RNU was performed in 78.8% of cases, with endoscopic disinsertion performed in most of them. Intravesical instillation of chemotherapy was applied to 3.5% of the patients, 1.8% received neoadjuvant chemotherapy, and 9.4% received adjuvant chemotherapy.

Most upper urothelial tumours were pT1 and high-grade (34.7%), and 15.9% were associated with CIS. Lymphadenectomy was not performed in the majority of patients, and 77.6% had free margins and 10% had lymphovascular involvement.

**Figure 1:**
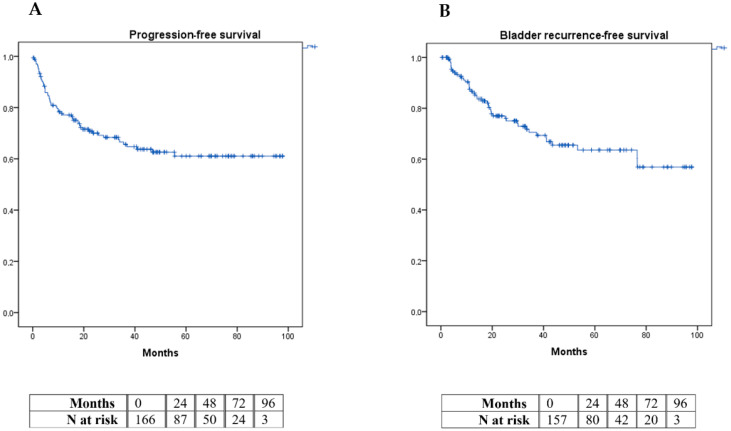
Kaplan–Meier curve graphic for progression-free survival (**A**) and intravesical recurrence-free survival (**B**).

A total of 32.9% of patients had a disease progression during follow-up, with local progression occurring in 19.6%, nodal progression in 30.3%, visceral progression in 42.8%, and bone progression in 7.1% of these. A total of 25.9% of the total number of patients presented with posterior BC; 32.9% progressed in a mean time of 65.64 ± 3.44 months (Figure 1A) and 25.9% presented with IVR at 68.33 ± 3.59 months (Figure 1B).

### 3.2. Disease Progression Multivariate Analysis

We found the statistically significant risk factors for PFS to be pT (HR 2.9, 95%CI 1.54–5.48, *p* = 0.01), margin involvement (HR 2.89, 95%CI 2.88–57.68, *p* = 0.01), and lymphovascular involvement (HR 7.97, 95% CI 1.43–44.42, *p* = 0.01) (Table 2).

#### IVR Multivariate Analysis

Having previously presented with BC (HR 3.736, 95% CI 1.24–11.22, *p* = 0.01) and the non-invasive appearance of the tumour on the CT (HR 0.229, 95% CI 0.05–0.95, *p* = 0.03) were significant risk factors for IVR, with the presence of hydronephrosis, tumour size, and lymphovascular invasion showing a non-significant trend (Table 3).

## 4. Discussion

Despite the advances in the treatment of UTUC, bladder recurrence and disease progression rate have not improved considerably over time. In our series, 32.9% patients experienced progression over a mean period of 65.64 months, which is comparable to what has been reported in other series [5]. Therefore, it seems appropriate to better characterise common risk factors for progression to better stablish follow-up protocols and intensify the management of high-risk patients.

Regarding the clinicopathological characteristics of the patients, we did not find any variables significantly associated with the risk of progression. However, Rojas et al. concluded that patients with a history of smoking, and with invasive tumours larger than 2 cm, have higher mortality [1,8]. Other studies describe pT stage (HR 25.58, 95% CI 9.854–66.445, *p* < 0.001) and histological grade (HR 1.697, 95% CI 1.100–2.617, *p* = 0.017), but not the location and clinical positive nodes, as prognostic factors for mortality [9]. Likewise, in our series, pT stage appears to be a statistically significant risk factor for PFS (HR 2.9, 95%CI 1.54–5.48, *p* = 0.01), but not histological grade. Other factors found to be statistically significant risk factors for disease progression in our cohort were margin involvement (HR 2.89; 95% CI 2.88–57.68, *p* = 0.01) and lymphovascular involvement (HR 7.970, 95% CI 1.43–44.42, *p* = 0.01).

Lee et al. showed that delaying RNU for more than three months was associated with poor overall survival, therefore a delay between ureteroscopy (URS) and RNU should not exceed this period [10]; however, in our case, surgical delay was not a significant factor to predict progression.

In a recent retrospective analysis led by Tuderti G et al., involving a large cohort, the role of neoadjuvant chemotherapy was highlighted in improving cancer-specific survival and overall survival (OS) in patients with cT ≥ 3 and positive cN [11]. Regarding adjuvant chemotherapy (AC), the recent POUT11 study concluded that in non-metastatic patients undergoing RNU with pT2-4 or pN+, AC improved recurrence-free survival (RFS) (HR 0.45, 95% CI 0.30–0.68; *p* = 0.0001) [12]. Consistent with this, Lo et al. performed a retrospective analysis of 245 patients where they proposed that AC is beneficial for OS and disease-free survival (DFS) [13]. Similar to previous results, Li et al. showed that the administration of AC improves OS in patients with locally progressed UTUC, particularly in lymph-node-positive individuals [14]. Correspondingly, a recent study concluded that AC significantly reduces cancer-specific mortality in lymph-node-positive (N1-2) patients, but not in lymph-node-negative (N0) patients, across all T stages from T2 to T4 [15].

On the other hand, the majority of patients who develop IVR do so during the two years following RNU, and the tumours are usually low-grade, multiple, papillary-like tumours, diagnosed as non-muscle-invasive BC [16]. In our series, 25.9% of patients presented with IVR at 68.33 ± 3.59 months. The majority of them resulted in pT1 (53.4%) or pTa (32.5%), and 74.4% corresponded to a high grade.

Some studies have concluded that tumour stage, URSs associated with preoperative biopsy, a previous history of BC, smoking, tumour location, endoscopic ureteral disinsertion, positive margins, surgical approach, and lymphovascular invasion are independent risk factors for IVR after RNU [1,17,18,19]. The study by Zhao et al. concluded that abnormal pre-surgical cytologies (HR = 3.101. 95%CI, 1.503–6.398, *p* = 0.002), hydronephrosis (HR = 1.852. 95%CI, 1.022–3.356, *p* = 0.042), AC (HR = 0.242. 95%CI, 0.123–0.437, *p* < 0.001), and a previous history of BC (HR = 5.51. 95%CI, 2.050–14.811, *p* < 0.001) are factors associated with IVR [20]. This finding is consistent with our results, since having previously presented with BC (HR 3.736, 95%CI 1.24–11.22, *p* = 0.019) turned out to be a statistically significant risk factor for IVR in the multivariate analysis, as was the non-invasive appearance of the tumour on the CT (HR 0.229, 95% CI 0.05–0.95, *p* = 0.034), but not smoking status or chemotherapy. In accordance with our findings, the meta-analysis by Seisen et al. also demonstrated that having had previous BC (HR 1.96, 95% CI 1.73–2.22; *p* < 0.001) is a significant predictor of IVR [21].

The multifocality of UTUC has been established in the literature as an important predictor of IVR in patients undergoing RNU; however, in our series, it did not appear to be a risk factor [19]. Other variables such as the presence of hydronephrosis, tumour size, and lymphovascular invasion showed a trend, although not significant.

There is an ongoing debate as to whether metachronous bladder tumours should be considered recurrences or second tumours. However, recent evidence supports the hypothesis that IVRs are mostly clonally related recurrences, suggesting that they correspond to the seeding of initial upper tract tumours [22]. In addition, recent studies have been carried out suggesting that URS and biopsy significantly increase the risk of IVR after RNU, due to direct manipulation of the tumour [23]. The current guidelines recommend using diagnostic URS (preferably without biopsy) only if imaging and/or urine cytology are not sufficient for the diagnosis and/or risk stratification of patients with suspected UTUC [22]. According to these statements, we evaluated URS as a risk factor for IVR in our cohort and could not find any associations.

On the other hand, the application of intravesical chemotherapy after RNU is also a relevant factor for IVR, and there are currently multiple studies evaluating it [24,25]. Furthermore, there are several studies that provide indirect evidence for the use of a dose of intravesical chemotherapy immediately after URS [26]; however, the limited number of patients who received this therapy does not allow us to reach any conclusion in our cohort.

Our results should be interpreted with caution. Apart from the retrospective nature of this study, limitations to consider include the limited use of perioperative systemic and intravesical chemotherapy (although this is now an established treatment; up to this point, we had only 16 cases of patients who had undergone adjuvant treatment, 6 patients who had undergone neoadjuvant treatment, and 3 who had undergone post-intravesical chemotherapy), as well as the heterogeneity and low number of lymphadenectomies performed (only 6 patients underwent lymphadenectomy), which does not allow us to conduct a scientifically valid study on the association between these treatments and prognosis. Additionally, the limited number of diagnostic URS procedures and the heterogeneity in distal ureteral management should also be taken into account as limitations. However, the multi-centre approach, together with the significant number of patients studied, confer valuable external validity to our results and allow us to add further evidence for risk factors associated with IVR and the disease progression of UTUC.

In our series, pT stage, margin involvement, and lymphovascular involvement were independent risk factors for UTUC progression. The fundamental risk factor for presenting with IVR after UTUC surgery was a previous history of BC.

## Figures and Tables

**Table 1:** Descriptive analysis of clinical and demographic cohort characteristics.

**Treatment**		
Approach	- Open surgery: 75 (44.1%) - Laparoscopic surgery: 90 (52.9%) - Not reported: 5 (2.9%)	
Distal ureter resection	- Clip ureter: 21 (12.3%) - Endoscopic disinsertion: 72 (42.3%) - Bladder cuff excision: 24 (14.1%) - Not reported: 53 (31.2%)	
Surgical technique	- Nephrectomy: 31 (18.2%) - RNU: 134 (78.8%) associated cystectomy: 11 (6.5%) - Ureterectomy: 1 (0.6%) - Endoscopic treatment: 4 (2.3%)	
Intravesical chemotherapy after RNU	- No: 164 (96.5%) - Yes: 6 (3.5%)	
Neoadjuvant chemotherapy	- No: 164 (96.5%) - Yes: 3 (1.8%) - Not reported: 3 (1.8%)	
Adjuvant chemotherapy	- No: 144 (84.7%) - Yes: 16 (9.4%) - Not reported: 10 (5.9%)	
**Pathological Anatomy**		
pT	- pTa	17 (10%)
	- pT1	65 (38.2%)
	- pT2	27 (15.9%)
	- pT3	45 (26.5%)
	- pT4	11 (6.5%)
	- pTis	2 (1.2%)
	- Not reported	3 (1.8%)
Associated CIS	- No: 141 (82.9%) - Yes: 27 (15.9%) - Not reported: 2 (1.2%)	
Grade	- High grade: 148 (87%) - Low grade:18 (10.6%) - Not reported: 4 (2.3%)	
pN	- Nx: 164 (96.5%) - N1: 4 (2.3%) - N2: 2 (1.2%)	
Margins	- Free: 132 (77.6%) - Affected: 30 (17.6%) - Not valuable: 8 (4.7%)	
Lymphovascular involvement	- No: 137 (80.6%) - Yes: 17 (10%) - Indeterminate: 16 (9.4%)	
Ureter involvement	- No: 69 (40.6%) - Yes: 87 (51.2%) - Not reported: 14 (8.2%)	
**Disease progression/IVR**		
Disease progression (excluding IVR)	- No: 112 (65.9%) - Yes: 56 (32.9%) - Not reported: 2 (1.2%)	
Type of progression	- Local: 11 (19.6% of all patients with progression) - Nodal: 17 (30.3% of all patients with progression) - Bone: 4 (7.1% of all patients with progression) - Visceral: 24 (42.8% of all patients with progression)	
IVR	- No: 126 (74.1%) - Yes: 44 (25.9%)	

All percentages shown in Table 1 were calculated based on the total cohort (170 patients), except for the type of progression, which was calculated based on the total number of patients who experienced progression. RNU (nephroureterectomy). CIS (carcinoma in situ). IVR (intravesical recurrence).

**Table 2:** Multivariate associative analysis for disease progression.

Variable	HR (Lower CI–Higher CI)	*p* Value
Sex	0.519 (0.139–1.944)	0.33
Age; years	0.961 (0.910–1.015)	0.15
CT size; cm	1.278 (0.856–1.908)	0.23
CT hydronephrosis; no/yes	1.743 (0.841–3.613)	0.13
pT (Ta < T1 < T2 < T3 < T4)	2.907 (1.542–5.480)	0.00
Grade (G1 < G2 < G3)	0.200 (0.010–3.997)	0.29
Associated CIS; no/yes	0.239 (0.045–1.275)	0.09
Affected margins; no/yes	12.895 (2.883–57.689)	0.00
Lymphovascular involvement; no/yes	7.970 (1.430–44.422)	0.01

Further adjusted by Smoker status, BMI, presurgical Hb, tumour location, surgery delay, and CT aspect. pT stage and grade were studied as ordinal variables because there is an increase associated with higher grades (1, 2, 3) or pT stages. BMI (body mass index). Hb (haemoglobin). CT (computed tomography scan). CIS (carcinoma in situ).

**Table 3:** Multivariate analysis for intravesical recurrence.

Variable	HR (Lower CI–Higher CI)	*p* Value
Sex	0.994 (0.331–2.988)	0.99
Age; years	0.978 (0.941–1.017)	0.25
Previous BC; no/yes	3.736 (1.244–11.225)	0.01
CT size; cm	1.405 (0.966–1.045)	0.07
CT hydronephrosis; no/yes	0.396 (0.148–1.061)	0.06
CT aspect; non-invasive/invasive	0.229 (0.055–0.956)	0.04
Multifocality; no/yes	0.943 (0.345–2.576)	0.90
Previous diagnostic URS; no/yes	1.360 (0.461–4.013)	0.57
pT (Ta < T1 < T2 < T3 < T4)	1.450 (0.936–2.246)	0.09
Associated CIS; no/yes	0.378 (0.101–1.420)	0.15
Affected margins; no/yes	1.683 (0.468–6.057)	0.42
Lymphovascular involvement; no/yes	4.825 (0.951–24.465)	0.05

Further adjusted by Smoker status, BMI, and approach. pT stage was analysed as an ordinal variable due to the increase associated with a higher pT stage. BMI (body mass index). BC (bladder cancer). CT (computed tomography scan). URS (ureteroscopy). CIS (carcinoma in situ).

## Data Availability

The original contributions presented in the study are included in the article/Supplementary Materials, further inquiries can be directed to the corresponding author.

## Supplementary Materials

The following supporting information can be downloaded at: https://www.mdpi.com/article/10.3390/diagnostics14222491/s1, Table S1: Multivariate analysis for disease progression; Table S2: Multivariate analysis for intravesical recurrence.

## Abbreviations

AC	Adjuvant chemotherapy
ASA	American Society of Anesthesiologists classification risk
BC	Bladder cancer
BMI	Body mass index
CIS	Carcinoma in situ
CT	Computed tomography
DFS	Disease-free survival
IVR	Intravesical recurrence
RNU	Nephroureterectomy
NMR	Nuclear magnetic resonance
OS	Overall survival
PFS	Progression-free survival
URS	Ureteroscopy
UTUC	Urothelial carcinoma of the upper urinary tract
