# Supplementary material for: Multi-Centre Study of Progression Factors and Intravesical Recurrence in Patients with Urothelial Carcinoma of the Upper Urinary Tract
Source: Diagnostics (Basel). 2024 Nov 7;14(22):2491. doi: 10.3390/diagnostics14222491 (PMC11593072; doi:10.3390/diagnostics14222491)
Supplement: Supplementary file 1 [file diagnostics-14-02491-s001.zip › diagnostics-3253288-supplementary.pdf]

Supplementary Materials

The tables corresponding to the comprehensive multivariate study of progression and intravesical recurrence are attached.

Variable		HR	Low	High	p Value
Sex		0.519	0.139	1.944	0.33
Age		0.961	0.910	1.015	0.15
Smoker or non smoker	Smoker	0.943	0.282	3.157	0.92
	Former smoker	0.363	0.077	1.723	0.20
BMI		0.991	0.877	1.119	0.87
Hb pre-surgical		0.844	0.617	1.154	0.28
CT aspect		0.217	0.41	1.153	0.07
CT size		1.278	0.856	1.908	0.23
Location	Pyelocalyceal	1.157	0.263	5.094	0.84
	Distal ureter	3.197	0.408	25.024	0.26
	Middle ureter	1.890	0.267	13.385	0.52
	Proximal ureter	7.231	0.414	126.410	0.17
	Multifocal	0.675	0.051	8.897	0.76
CT hydronephrosis		1.743	0.841	3.613	0.13
Delay until surgical treatment		0.999	0.995	1.004	0.80
pT (Ta<T1<T2<T3<T4)		2.907	1.542	5.480	0.00
Grade		0.200	0.010	3.997	0.29
Associated CIS		0.239	0.045	1.275	0.09
Affected margins		12.895	2.883	57.689	0.00
Lymphovascular involvement		7.970	1.430	44.422	0.01

Supplementary Table S1. Multivariate analysis for disease progression

BMI (body mass index). Hb (hemoglobin). CT (computed tomography scan). CIS (carcinoma in situ).

Variable		HR	Low	High	p Value
Sex		0.994	0.331	2.988	0.99
Age		0.978	0.941	1.017	0.25
Smoker or non smoker	Smoker	1.678	0.557	5.060	0.35
	Former smoker	0.930	0.312	2.768	0.89
BMI		1.007	0.916	1.108	0.88
Previous BC		3.736	1.244	11.225	0.01
CT size		1.405	0.966	1.045	0.07
CT hydronephrosis		0.396	0.148	1.061	0.06
CT aspect		0.229	0.055	0.956	0.04
Multifocality		0.943	0.345	2.576	0.90
Previous diagnostic URS		1.360	0.461	4.013	0.57
Approach		0.985	0.397	2.443	0.97
pT (Ta<T1<T2<T3<T4)		1.450	0.936	2.246	0.09
Associated CIS		0.378	0.101	1.420	0.15
Affected margins		1.683	0.468	6.057	0.42
Lymphovascular involvement		4.825	0.951	24.465	0.05

Supplementary Table S2. Multivariate analysis for intravesical recurrence.

BMI (body mass index). BC (bladder cancer). CT (computed tomography scan). URS (ureteroscopy). CIS (carcinoma in situ).
